# Multimodal prehabilitation and postoperative outcomes in upper abdominal surgery: systematic review and meta-analysis

**DOI:** 10.1038/s41598-024-66633-6

**Published:** 2024-07-11

**Authors:** Farshad Amirkhosravi, Kelvin C. Allenson, Linda W. Moore, Jacob M. Kolman, Margaret Foster, Enshuo Hsu, Farzan Sasangohar, Atiya Dhala

**Affiliations:** 1 Department of Surgery, Houston Methodist, Houston, TX USA; 2 Office of Faculty and Research Development, Department of Academic Affairs, Houston Methodist, Houston, TX USA; 3 School of Medicine, Department of Medical Education, Texas A&M University, College Station, TX USA; 4 Center for Health Data Science and Analytics, Houston Methodist, Houston, TX USA; 5 Wm Michael Barnes ‘64 Department of Industrial and Systems Engineering, Texas A&M University, College Station, TX USA; 6 Center for Critical Care, Houston Methodist, Houston, TX USA

**Keywords:** Rehabilitation, Lifestyle modification, Nutrition, Outcomes research

## Abstract

The impact of multimodal prehabilitation on postoperative complications in upper abdominal surgeries is understudied. This review analyzes randomized trials on multimodal prehabilitation with patient and hospital outcomes. MEDLINE, Embase, CINAHL, and Cochrane CENTRAL were searched for trials on prehabilitation before elective (non-emergency) abdominal surgery. Two reviewers independently screened studies, extracted data, and assessed study quality. Primary outcomes of interest were postoperative pulmonary complications (PPCs) and all-cause complications; secondary outcomes included hospital and intensive care length of stay. A meta-analysis with random-effect models was performed, and heterogeneity was evaluated with I-square and Cochran’s Q test. Dichotomous variables were reported in log-odds ratio and continuous variables were presented as mean difference. Ten studies (total 1503 patients) were included. Odds of developing complications after prehabilitation were significantly lower compared to various control groups (− 0.38 [− 0.75– − 0.004], *P* = 0.048). Five studies described PPCs, and participants with prehabilitation had decreased odds of PPC (− 0.96 [− 1.38– − 0.54], *P* < 0.001). Prehabilitation did not significantly reduce length of stay, unless exercise was implemented; with exercise, hospital stay decreased significantly (− 0.91 [− 1.67– − 0.14], *P* = 0.02). Multimodal prehabilitation may decrease complications in upper abdominal surgery, but not necessarily length of stay; research should address heterogeneity in the literature.

## Introduction

More than four of 10 adults over the age of 60 in the United States will undergo intra-abdominal surgery during their lifetime^1^, 30% of whom are expected to experience postoperative complications. Major abdominal surgeries are often complex, involving extensive incisions, manipulations of intra-abdominal internal organs, and digestive resections with reconstruction via anastomosis or stoma creation^2^.

Patients undergoing major abdominal surgery, especially upper abdominal surgery, are predisposed to postoperative pulmonary complications (PPCs)^3–6^ due to compromised pulmonary mechanics caused by multiple factors. First, the residual effects of combined anesthetic and neuromuscular blocking agents result in hypoventilation^7^. Second, these extensive laparotomy incisions extend to the sternum causing splinting of the diaphragm from pain, accompanied by shallow breathing and inadequate cough, leading to retention of secretions and subsequently atelectasis and pneumonia^8,9^. These factors contribute to postoperative respiratory failure—a combination of hypoxemic and hypercapnic respiratory failure^4,10,11^.

These outcomes delay return to normal quality of life^12^, exacting a psychosocial toll on patient-family units^13^, while placing a significant financial burden on healthcare systems^14^. Even without postoperative complications, up to 40% of patients having major abdominal surgery experience reduced physical function and lower quality of life^15–18^. Frailty, sarcopenia, and malnutrition measurably contribute to poor outcomes^19–22^. Targeting these risk factors pre-surgery may reduce PPCs and improve overall patient outcomes.

In recent years, efforts to improve postoperative outcomes have predominantly focused on intraoperative and postoperative management^23–25^. Conversely, a preoperative approach, prehabilitation, emphasizes the preoperative optimization of patient-centered risk factors such as malnutrition and reduced functional capacity^26,27^, augmenting established enhanced recovery after surgery (ERAS) pathways^28,29^. The concept of prehabilitation emerged in the 1940s to describe the physical conditioning of soldiers for military services^30^. The topic re-emerged in the 1980s in its modern medical context^31^. “Prehab” programs have spread from initial use in sports medicine to include multiple modalities apart from physical exercise alone, and multiple populations, notably patients with cancer and patients undergoing major abdominal surgery^31^. For these populations, there is a window of opportunity to proactively identify and address patient-centered factors during the time leading up to planned surgery, or during neoadjuvant treatment in the setting of cancer.

Multimodal prehabilitation is designed to improve recovery time following elective surgery by optimizing physical, nutritional, psychiatric and other comorbid conditions of a patient undergoing surgery^26^. A multimodal prehabilitation program offers comprehensive preparation to improve outcomes, especially for the growing population of older adults (> 65 years) diagnosed with gastrointestinal, gynecological, and urological cancer, for whom surgery remains the mainstay treatment despite a predisposition to frailty, sarcopenia, and poor postoperative outcomes^32,33^. Prehabilitation could be essential to improve eligibility and recovery chances for patients who would not otherwise qualify for surgery.

Studies have aimed to qualify the potential impact of prehabilitation on peri- and postoperative outcomes. Many of these studies suffer from heterogeneity of participants, programs, planned type of surgery, and outcomes of interest^34–38^. While the diversity of individual trials limits generalizability, targeted studies have shown statistically significant benefits of certain prehabilitation programs^35,39–41^, including functional capacity^36^. Despite limitations of current evidence, clinicians readily identify patient-centered risk factors that portend poor surgical outcomes and have sought in good faith to optimize or correct these risk factors within the limits of available preoperative timeframes.

Like individual studies, meta-analyses do not focus on a consistent scope for prehabilitation or consistently explore multimodal prehab specifically. Some reviews^37,38,42^ include both cohort studies and randomized controlled trials (RCTs), while others^34–36,39–41^ restrict scope to RCTs. Of the systematic reviews restricted to RCTs, some^35,39^ examine prehabilitation solely involving physical activity. Of the research including multimodal prehabilitation^36,37,40,41^, prior systematic reviews even on nominally “abdominal” procedures have been broad, including procedures focused both below and above the diaphragm including esophageal procedures^37,40,41^. Lower abdominal surgical procedures have a markedly lower risk factor for PPCs compared to upper abdominal surgery, which alters lung mechanics more readily due to its proximity to the diaphragm^10^, whereas esophageal surgery is linked to higher postoperative pulmonary morbidity^43^. Combining lower abdominal, upper abdominal, and esophageal surgery exacerbates heterogeneity, likely obscuring the linkage between the prehabilitation intervention and postoperative complications. No review of prehabilitation has focused on complex upper abdominal surgeries with impacts on PPCs as a key outcome.

The objective of this systematic review was to examine RCTs to demonstrate the impact of preoperative interventions on postoperative clinical and patient-related outcomes, answering the primary question: in patients who are frail and having elective major upper abdominal surgery, including hepatopancreaticobiliary surgery, does prehabilitation reduce PPCs and other complications compared to patients who do not undergo prehabilitation?

## Methods

This report complies with the preferred reporting items for systematic reviews and meta-analyses (PRISMA 2020)^44^ and assessing the methodological quality of systematic reviews (AMSTAR-2)^45^ guidelines. Additional protocol details and data sheets are available upon reasonable request; the protocol was registered^46^ retrospectively due to a mid-extraction amendment to change from a scoping review (less commonly registered) to a systematic review once meta-analysis was confirmed to be feasible.

### Search strategy

The search strategy was developed by a librarian specializing in systematic reviews (MF). On February 22, 2022, MF retrieved records from MEDLINE (Ovid), Embase (Ovid), CINAHL (EBSCOhost), and Cochrane CENTRAL, using filters for date range, human study RCTs, and exclusion of retracted material per best practices for Ovid interfaces^47^ and CINAHL^48^. For a list of search strings used for each database, see Supplementary File: search strategy.

### Inclusion and exclusion criteria

Studies were included according to the PICO (population, intervention, comparator, outcome) rubric^49^. The population of interest comprised adult patients with sarcopenia or frailty^50^ undergoing elective (i.e., non-emergency) hepatopancreaticobiliary surgery, including exploratory laparotomy, pancreatectomy, distal pancreatectomy, pancreaticoduodenectomy, Whipple procedure, and pancreatic enucleation; elective abdominal surgeries; elective major abdominal surgeries; elective complex abdominal surgeries; elective abdominal oncologic surgeries; elective hepatobiliary surgery; open hepatectomies; open elective colon surgeries; and open elective gastric resection. Any mode of prehabilitation intervention was included—physical, nutritional, psychiatric, or other co-morbid condition optimization designed to improve recovery time—compared to patients not receiving prehabilitation in an RCT design. Non-RCTs were excluded to mitigate concerns regarding heterogeneity and bias which can occur in mixed-methods reviews^51^. Studies not reporting results relevant to our primary outcomes of interest, i.e., related to oxygenation or pulmonary function or other postoperative complication broadly construed (see below), were excluded (e.g., protocols).

Primary RCT reports from the year 2000 to search date were included with no language restriction. The date range was selected because rigorous primary studies on prehabilitation started in the early 2000s^52,53^, which is also when the concept of frailty had been most explicitly defined^50^. Peer-reviewed journal articles were included. Non-RCT study designs, unpublished work, technical reports, conference materials, and preprints were excluded.

After automated removal of duplicates using Covidence^54^, two reviewers (AD and FA) independently screened titles and abstracts for inclusion; these reviewers also assessed full text eligibility, using Covidence to streamline processing. Raters met for discussion as needed to assess disagreements and reach consensus at each step.

### Data extraction

Data were collected on: setting (country) of study; study aim; period (dates, if reported); timing of prehabilitation (number of days/weeks before surgery, variations by treatment/comparator groups when relevant); population description (n-counts, inclusion/exclusion criteria) and demographics by group, including age, race/ethnicity, and gender; recruitment setting; modality of prehabilitation (physical therapy, nutritional therapy, psychosocial; anemia-related; respiratory therapy); primary and secondary outcome descriptions (retained for synthesis and meta-analysis if deemed feasible by the team’s statistician, EH); and funding source and author declarations of potential conflicts of interest. For screening, two raters independently charted information and, using side-by-side comparison of extractions in Covidence, met to form consensus.

### Outcomes of interest

Our two primary outcomes of interest were PPCs and all-cause complications—inclusive of acute lung injury, postoperative pneumonia, or other postoperative pulmonary complications (aspiration, respiratory failure including hypoxic), non-invasive ventilation, unplanned intubation (re-intubation after postoperative extubation), and respiratory failure. Secondary outcomes included length of stay (LOS) in the hospital or intensive care unit (ICU).

### Quality assessment

For risk of bias assessment, the JBI critical appraisal checklist for RCTs^55^ was used. As for the screening process above, two raters independently applied the JBI instrument and then compared results and met for discussion to reach consensus, as facilitated by side-by-side comparison views in the Covidence platform. Studies were not excluded based on appraisal. In the case of prehabilitation studies, we anticipated that blinding participants and interventionalists would frequently and justifiably not be feasible.

### Statistical analysis

Regarding effect measures for mortality, PPCs, and other complications, log-odds ratio is reported; for functional capacity, hospital LOS, and ICU LOS, mean difference is reported below. For continuous outcomes reported as median (IQR), we estimated mean and standard deviation following Wan et al.^56^.

For synthesis of each outcome, we report a table and a forest plot with summary statistics (log-odds ratio or mean difference) of each study and overall results. We performed a meta-analysis with random-effect models. Heterogeneity was evaluated with I-square (%) following Cochrane^57^, by which 0–40% can be interpreted as a possibly unimportant level of inconsistency; 30–60% as moderate heterogeneity; 50–90% as substantial heterogeneity; and 75–100% as considerable heterogeneity (ranges overlap as interpretation of meaningful inconsistency also depends upon factors such as effect size and direction as well as strength of evidence, e.g., *P* value).

Publication bias was evaluated with funnel plots for primary outcomes. Studies that fell outside of the 95% CI were considered as having potential bias. For certainty assessment, heterogeneity was evaluated with I-square. We performed subgroup analysis by stratifying prehab method (exercise vs. no exercise). When potential publication bias was detected, we performed sensitivity analysis by removing studies with potential publication bias. All statistical analysis was performed with Stata 16.1 (StataCorp LLC, College Station, Texas, USA).

### Ethics declarations

Ethics committee review and participant consent are not applicable (literature review and meta-analysis); no trademarked drugs, chemicals, instruments, or other devices are discussed.

## Results

### Search results

**Figure 1:**
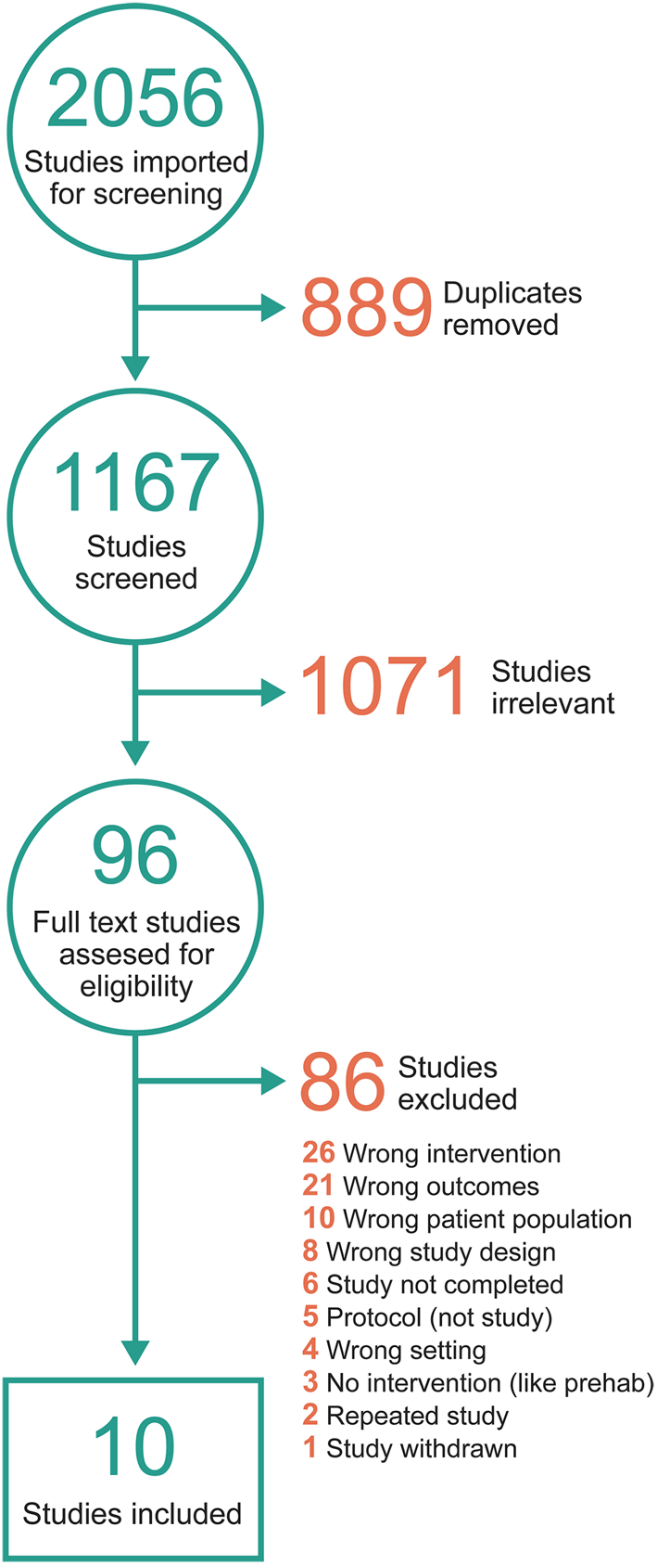
PRISMA flowchart of data selection.

The initial search yielded 1167 distinct studies for screening, of which 96 articles underwent full-text review; 10 articles were selected for this systematic review^58–67^. Additionally, the references of the included studies were searched, and the team conducted forward searching (review of references found to have later cited our included articles); however, no additional articles met inclusion criteria (Fig. 1).

### Characteristics of included studies

**Figure 2:**
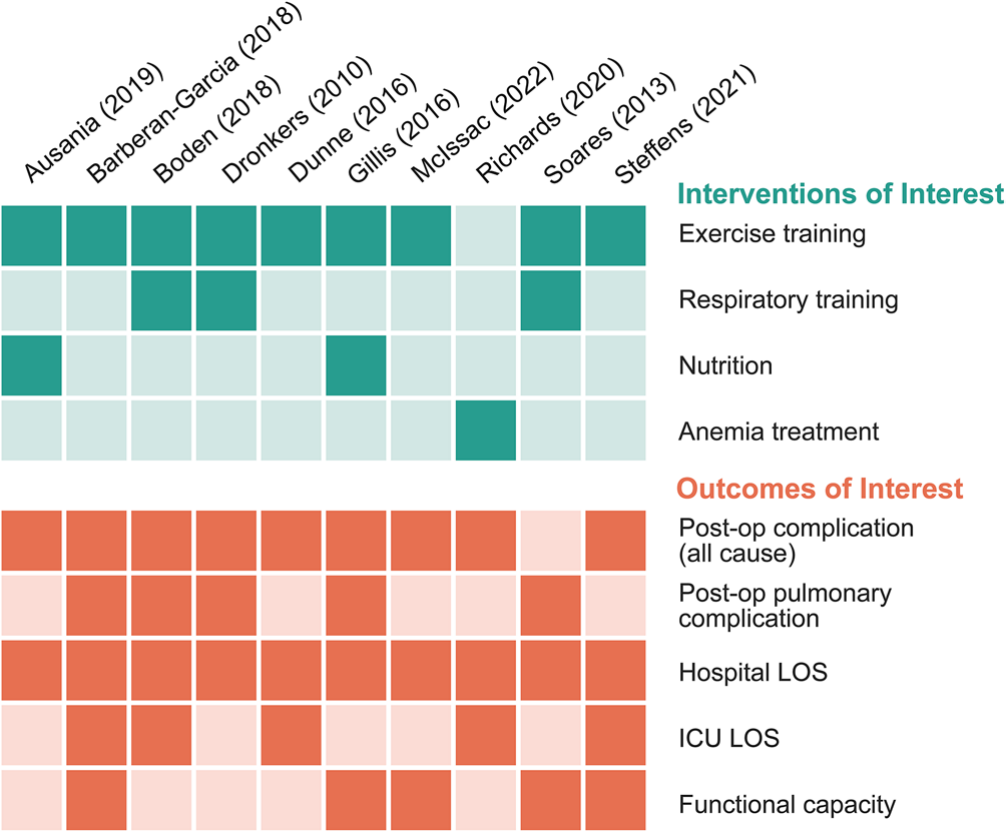
Intervention and outcome of interest of each study.

Participants in all 10 included studies were recruited in-office during their preoperative evaluation. The length of prehabilitation applied in the studies ranged from 8 days^62^ to 6 weeks^58,64^ (Table 1). Prehabilitation included exercise training (eight studies)^58,60–63,65–67^, respiratory training (three studies)^60,62,66^, nutrition support (two studies)^64,67^, and anemia treatment (one study)^59^. Three studies combined exercise and respiratory training^60,62,66^ and one study^67^ used exercise and nutritional optimization. Outcomes of interest included all cause complications^58–65,67^, respiratory complications^60–62,64,66^, LOS^58–67^, ICU LOS^58,59,61–63^, and functional capacity (Fig. 2)^58,61,64–66^. However, due to inconsistent definitions for functional capacity (e.g., walk tests measured as time versus distance), we excluded this planned outcome of interest from meta-analysis. Mortality was not reported widely enough for analysis.

**Table 1:** General characteristics^a^ of included studies.

First author (year)	Country	N-count	Age, intervention group	Age, control group	Gender (% female)	Length of prehab
Ausania (2019)	Spain	40	Median 66.1	Median 65.7	45	Mean 17 days
Barberan-Garcia (2018)	Barcelona, Spain	144	Mean 71 (SD 11)	Mean 71 (SD 10)	24.8	4 weeks
Boden (2018)	Australia and New Zealand	441	Median 63.4 (IQR 51.5–71.9)	Median 67.5 (56.3–75.3)	38.4	Median 8–9 days
Dunne (2016)	UK	38	Median 61 (IQR 56–66)	Median 62 (IQR 53–72)	29.7	4 weeks
Dronkers (2010)	Netherlands	42	Mean 71.1 (SD 6.3)	Mean 68.8 (SD6.4)	26.2	2–4 weeks
Gillis (2016)	Canada	48	Mean 67.6 (SD 11.5)	Mean 69 (SD 9.4)	34.9	6 weeks
McIsaac (2022)	Canada	204	Mean 74 (SD7)	Mean 74 (SD 6)	56.6	4 weeks
Richards (2020)	UK	487	Median 66 (IQR 57–72)	Median 65 (IQR 50–72)	54.8	10–42 days
Soares (2013)	Brazil	37	Median 58.5 (IQR 51.3–63.5)	Median 55.0 (IQR 49.3–64.3)	46.9	2 weeks
Steffens (2021)	Australia	22	Mean 62.0 (range 48.0–72.0)	Mean 66.0 (range 46.0–70.0)	45.5	2–6 weeks

^a^For more extensive clinical characteristics and outcome measures, see Supplementary Information file: Included_studies_outcome_measures.xlsx.

Among the patients who received some form of prehab modality, exercise intervention was the most predominant (n = 457) followed by anemia management (n = 244), exercise combined with inspiratory muscle training (n = 38), and nutrition (n = 24). In all, 734 patients received prehabilitation and contributed data for the meta-analysis (1503 including controls). Six of the 10 studies focused on cancer patients^58,60,63–65,67^ while the remaining four studies included non-cancer patients undergoing surgery for benign etiologies as well^59,61,62,66^. Compliance with prehab programs and enrollment rates were not widely characterized.

### Results of quality assessment

A summary of quality assessment (Supplementary Figures S1, S2) found eight of the 10 included studies lacked the ability to blind participants and the treatment providers, as anticipated in a physical prehabilitation setting. Of more substantive concern, one study^66^ did not blind the outcome assessors, while another^67^ was unclear about randomization methods, allocation concealment, and blinding of the outcome assessors. Additionally, one study^60^ had participant dissimilarities at baseline.

### Meta-analysis results

#### Postoperative pulmonary complications

**Figure 3:**
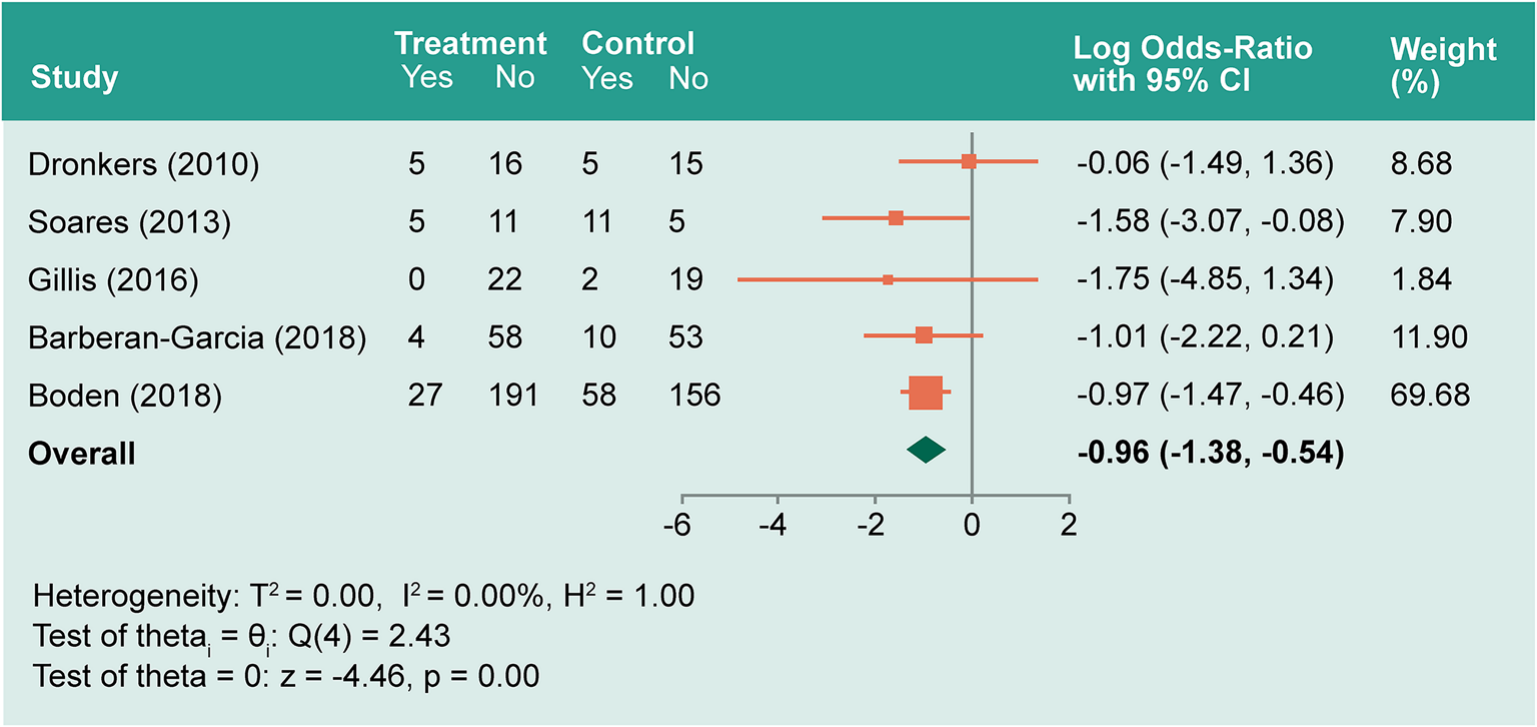
Forest plot showing pooled log odds ratio for pulmonary complication.

Five studies^60–62,64,66^ included PPCs. No significant heterogeneity (*I*^*2*^ ≈ 0.0%) or publication bias was observed (Supplementary Figure S3). Patients undergoing prehabilitation had a significantly decreased log odds-ratio compared to the control group by − 0.96 (− 1.38– − 0.54, *P* < 0.001; Fig. 3).

#### All-cause complications

**Figure 4:**
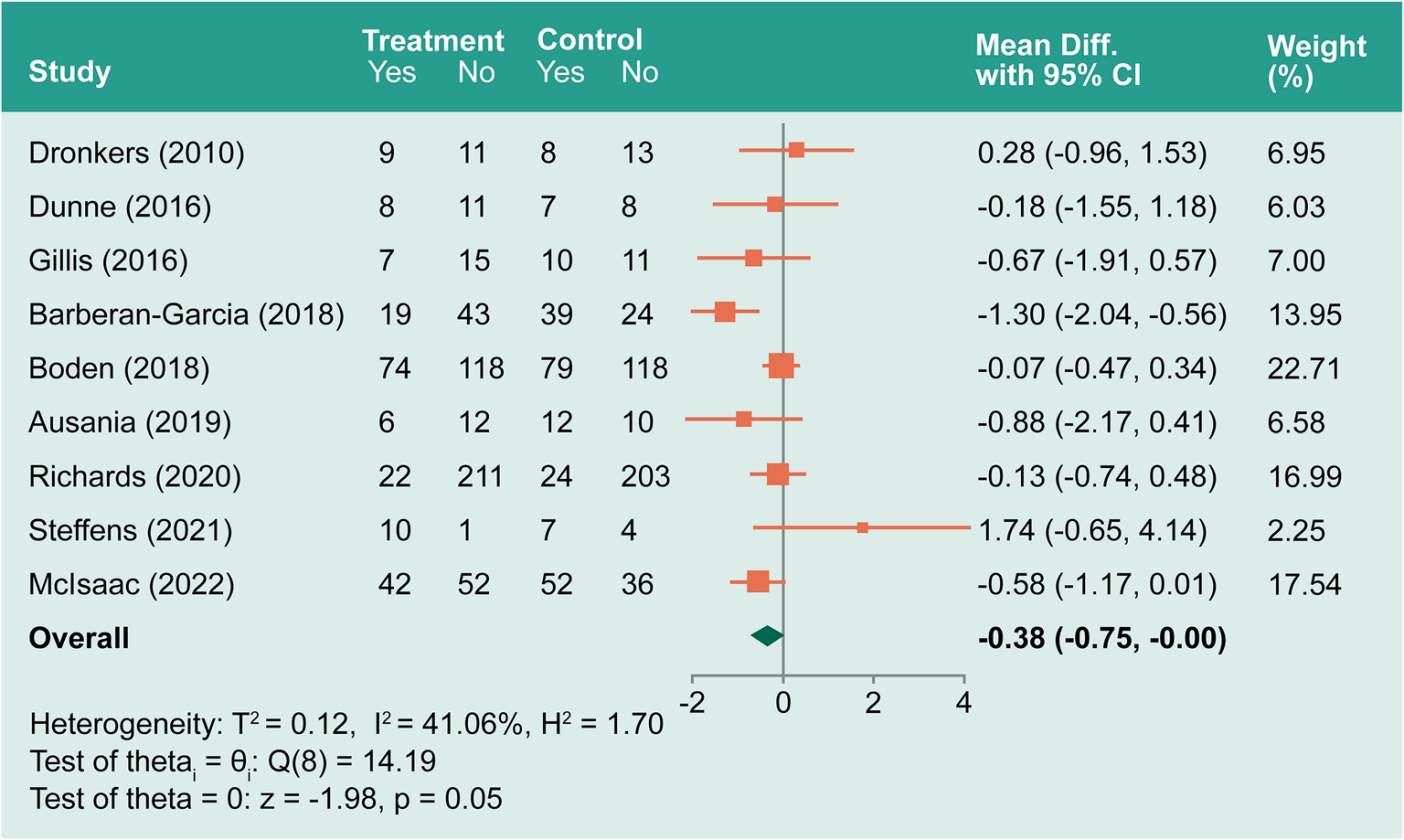
Forest plot showing pooled all cause complication.

Nine studies^58–65,67^ included all-cause complications. The random-effects maximum likelihood (REML) model showed moderate heterogeneity (*I*^*2*^ = 41.06%). The overall log odds-ratio was − 0.38 (− 0.75– − 0.004, *P* = 0.048; Fig. 4). However, the funnel plot suggests a potential publication bias. We performed sensitivity analysis by removing studies with potential publication bias and the log odds-ratio was − 0.21 (− 0.475–0.055, *P* = 0.1209). See Supplementary Files: Figure S4 and associated sensitivity analysis.

**Figure 5:**
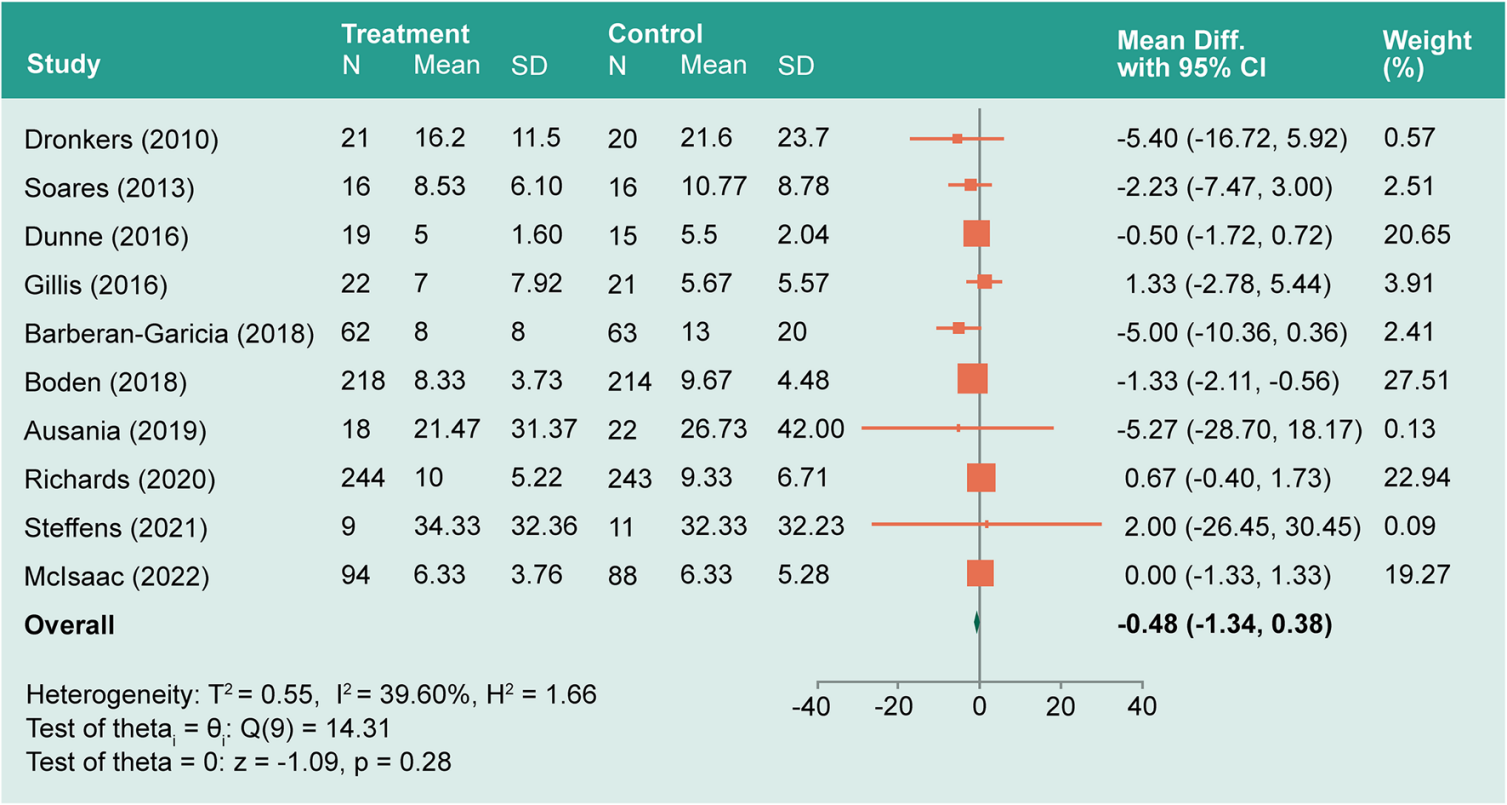
Forest plot showing pooled hospital length of stay.

**Figure 6:**
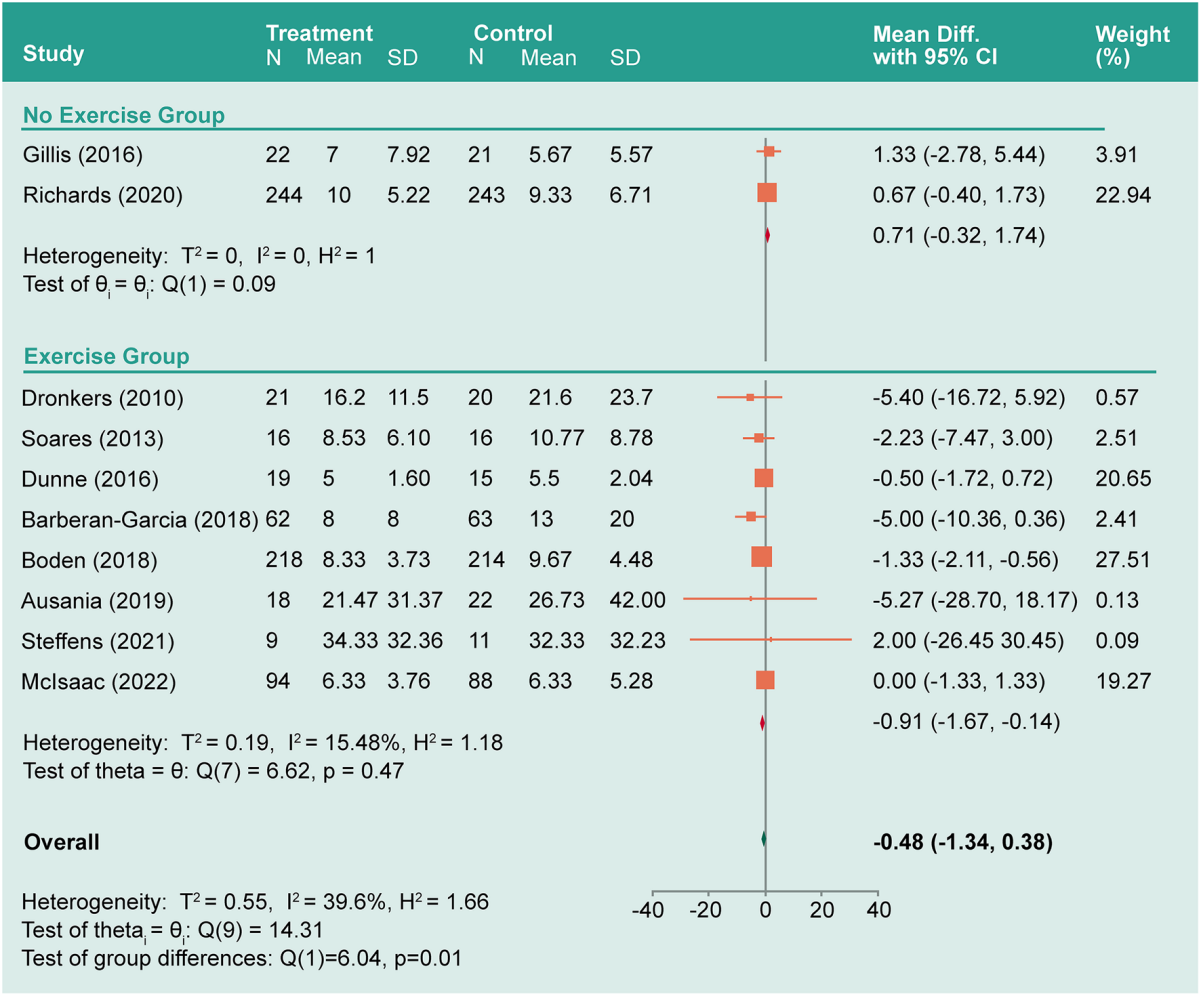
Forest plot showing pooled hospital LOS subgrouped by use of exercise method as prehabilitation.

*Hospital length of stay*: The hospital LOS was an outcome of interest in all included studies. The model showed moderate heterogeneity (*I*^*2*^ = 39.6%). The overall mean difference was − 0.48 between the prehabilitation group and the control group (− 1.34–0.38, *P* = 0.28; Fig. 5). Studies were subgrouped based on the prehabilitation methodology (exercise vs no exercise). In the exercise group, *I*^*2*^ decreased to 15.5% and the LOS mean difference was significantly decreased by − 0.91 (− 1.67– − 0.14, *P* = 0.02; Fig. 6). In the no exercise group, *I*^*2*^ decreased to ≈ 0.0% while the LOS mean difference was not significant 0.71 (− 0.33–1.74, *P* = 0.76). See also Supplementary Information for associated sensitivity analysis.

#### ICU length of stay

**Figure 7:**
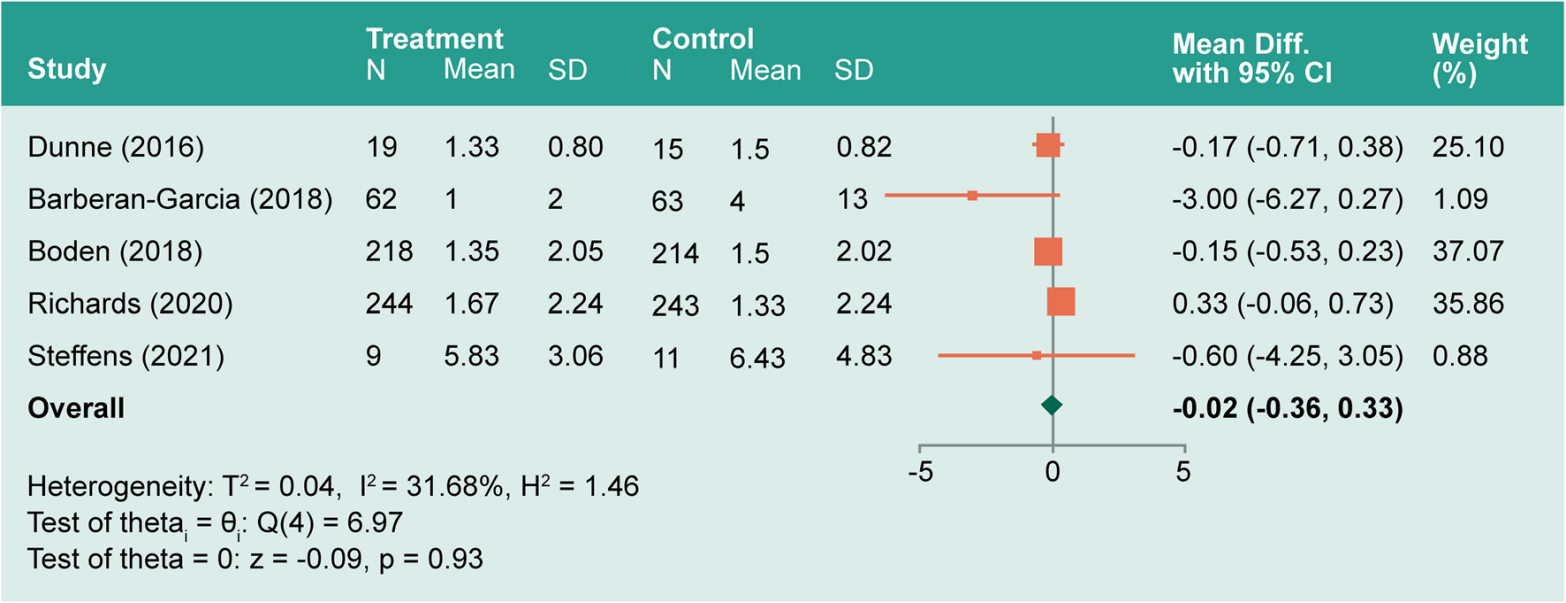
Forest plot showing pooled ICU length of stay.

ICU LOS was described in five studies^58,59,61–63^, with moderate heterogeneity (*I*^*2*^ = 31.68%). The overall mean difference between prehabilitation and control groups (− 0.02) was non-significant (− 0.36–0.33, *P* = 0.93; Fig. 7).

## Discussion

Our systematic review analyzed 10 high-quality RCTs with a total of 1503 patients to assess the impact of prehabilitation on postoperative outcomes in patients undergoing major upper abdominal surgeries.

The major finding of the present study was the identification of lower odds of developing PPCs among the prehabilitation group with no significant heterogeneity as well as a decrease in all-cause complications. It should be emphasized that postoperative pulmonary complications are a well-known risk of major abdominal and/or gastrointestinal cancer operations. In a large, multi-center trial by Fernandez–Bustamante and colleagues^68^, the occurrence of even one such complication was associated with increased mortality, ICU admission, and length of stay. Apart from the morbidity, these complications are also associated with increased medical costs ranging between $26,000 and $48,000 US dollars in recent studies^69,70^. Many such studies call for the implementation of prehabilitation-oriented interventions to help reduce such healthcare burdens. While minimally invasive surgical approaches have demonstrated significant potential to further decrease PPCs, its implementation in complex gastrointestinal and cancer operations is still emerging^71,72^. Our aggregate conclusion that preoperative interventions appear to provide protective benefits against postoperative complications was generally consistent with the conclusions in the majority of research studies reviewed and analyzed.

Although we specifically used a uniquely narrowed definition of our intervention of interest to reduce heterogeneity based especially in the scope of abdominal studies, there is room for some comparison. In different aspects, our study population most closely resembles the meta-analyses by Hijazi et al.^38^ (which included multiple modes of prehabilitation in major abdominal surgery, not restricted to RCTs) and Heger et al.^39^ (restricted to RCTs exploring physical prehabilitation only). Our results support findings by Heger and colleagues that prehab and specifically exercise significantly reduces PPCs; however, our finding of lower all-cause complications in the prehabilitation group with moderate heterogeneity in the meta-analysis contrasts from Heger et al., who did not find significant reduction in all-cause complications. Notably, Hijazi and colleagues did not identify a difference in postoperative complications between prehabilitation and standard of care groups, likewise citing heterogeneity at multiple levels. Hughes and colleagues^35^ examined patients undergoing major vascular and (laparoscopic) bariatric procedures, a major difference in population from our study, but similarly found a reduction in PPCs; the authors similarly selected data to reduce the heterogeneity and concluded that the potential postoperative benefits can be achieved with appropriate prehabilitation.

Our meta-analysis also examined hospital LOS, an outcome with moderate heterogeneity. Subgroup analysis comparing prehab exercise versus no exercise had a low heterogeneity (12–15%) with prehab patients demonstrating significantly lower hospital LOS (0.91 days). The no-exercise group did not have significant differences. Other systematic reviews demonstrate mixed results on hospital LOS, with one review focused on cancer patients showing encouraging results^41^ while others on older adults (including but not limited to cancer patients) found no differences^35,40,42^. We likewise found no significant reduction in ICU LOS (moderate heterogeneity) despite the promising findings on lower PPCs. Since the need and subsequent length of stay can differ between clinical populations and procedures, these findings may be particularly sensitive to heterogeneity and require focused, population-specific attention (age and procedure-specific) and cautious generalization.

Although Canada, Europe and the UK, Australia and New Zealand, and Brazil were represented among the studies included in our systematic review, no articles were from the United States or other major geographical areas including the Asian and African continents, broadly indicating a paucity of evidence on and lack of global generalizability for prehabilitation and the need for additional setting-specific studies.

### ERAS as a model

We concur with others that the field of prehabilitation needs standardization to reduce confounding and better distinguish benefits of specific interventions^73^ and produce generalizable research results. Obvious parallels exist between prehab and ERAS programs^74,75^, and we conclude that the ERAS Society provides an ideal model for advocates of prehab programs to improve standardization. The ERAS Society has published standards for the development of future guidelines to ensure that established findings continue to apply across individual guidelines and that recommendations between guidelines are not contradictory. These guidelines employ champions from all aspects of the care team. Similar strategies could be employed in scaling prehabilitation programs.

### Limitations

Despite inclusion of quality-assessed RCTs, our findings were limited by heterogeneity in the design, conduct, and reporting of the primary studies. We initially sought RCT reports with multimodal intervention modalities; however, most of the research focused on unimodal or bimodal prehab. We also discovered that frailty and sarcopenia were often omitted from the baseline characteristics among these studies, indicating a gap in reporting this salient feature which limited our ability to draw conclusions on our population of interest. We join other researchers^40^ in encouraging future primary studies to track and report this important characteristic of surgical patients. Lastly, our review may be limited by the exclusion of non-RCTs, a methodological choice on which opinions have differed and evolved^51^; future systematic review updates should consider inclusion of non-RCTs, particularly until additional, well-focused RCTs emerge in the literature.

## Conclusion

Despite the limitations, our systematic review and meta-analysis uniquely focuses on prehabilitation in several ways. We included RCTs in patients who required major elective open abdominal surgery with consistent durations of prehabilitation between 8 days and 6 weeks. We found heterogeneity to be a limiting factor in collecting data on outcomes, supporting the need for unified effort inclusive of all members of prehabilitation teams to standardize consensus protocols and statistical analysis. Prehab and ERAS programs should continue to function by identifying an at-risk population, implementing an evidence-based, personalized multimodal intervention, measuring the desired intervention benefits, and quantifying perioperative outcomes. Standardization of intermediate objective measures to quantify physiologic/physical responses to prehab are also needed. Lastly, while the current literature presents conflicted or weak support for prehab, we believe stratifying evidence-based components of prehab programs within specific disease sites, like the efforts of the ERAS Society, will reduce confounding and heterogeneity serving to create more generalizable results. Once prospective, disease-site specific prehab findings are available in a diversity of geographical settings, future meta-analyses will be able to support and validate potential bundled and multimodal prehabilitation care.

### Supplementary Information

Supplementary Information 1.

Supplementary Information 2.

## Data Availability

All data generated or analyzed during this study are included in this article and its supplementary material files. Further enquiries can be directed to the corresponding author.

## References

[1] Nunoo-Mensah JW, Rosen M, Chan LS, Wasserberg N, Beart RW (2009). Prevalence of intra-abdominal surgery: What is an individual’s lifetime risk?. South. Med. J..

[2] Straatman J, Cuesta MA, de Lange-de Klerk ESM, van Peet DL (2016). Long-term survival after complications following major abdominal surgery. J. Gastrointest. Surg..

[3] Patel K (2016). Postoperative pulmonary complications following major elective abdominal surgery: A cohort study. Perioper. Med..

[4] Jensen JH, Sørensen L, Mosegaard SB, Mechlenburg I (2022). Risk stratification for postoperative pulmonary complications following major cardiothoracic and abdominal surgery—Development of the PPC risk prediction score for physiotherapists clinical decision-making. Physiother. Theory Pract..

[5] Karim SAM, Abdulla KS, Abdulkarim QH, Rahim FH (2018). The outcomes and complications of pancreaticoduodenectomy (whipple procedure): Cross sectional study. Int. J. Surg..

[6] Katsura M, Kuriyama A, Takeshima T, Fukuhara S, Furukawa TA (2015). Preoperative inspiratory muscle training for postoperative pulmonary complications in adults undergoing cardiac and major abdominal surgery. Cochrane Database Syst. Rev..

[7] Karcz M, Papadakos PJ (2013). Respiratory complications in the postanesthesia care unit: A review of pathophysiological mechanisms. Can. J. Respir. Ther..

[8] Kelkar KV (2015). Post-operative pulmonary complications after non-cardiothoracic surgery. Indian J. Anaesth..

[9] Kim SH (2010). An evaluation of diaphragmatic movement by M-mode sonography as a predictor of pulmonary dysfunction after upper abdominal surgery. Anesth. Analg..

[10] Fernandes A (2019). Root causes and outcomes of postoperative pulmonary complications after abdominal surgery: A retrospective observational cohort study. Patient Saf. Surg..

[11] Drummond G (1999). Surgery and respiratory muscles. Thorax.

[12] Badia JM (2017). Impact of surgical site infection on healthcare costs and patient outcomes: A systematic review in six European countries. J. Hosp. Infect..

[13] Pinto A, Faiz O, Davis R, Almoudaris A, Vincent C (2016). Surgical complications and their impact on patients’ psychosocial well-being: A systematic review and meta-analysis. BMJ Open.

[14] Lindsay JO, Bergman A, Patel AS, Alesso SM, Peyrin-Biroulet L (2015). Systematic review: The financial burden of surgical complications in patients with ulcerative colitis. Aliment. Pharmacol. Ther..

[15] Buurman BM (2012). Clinical characteristics and outcomes of hospitalized older patients with distinct risk profiles for functional decline: A prospective cohort study. PLoS One.

[16] Welvaart WN (2011). Selective diaphragm muscle weakness after contractile inactivity during thoracic surgery. Ann. Surg..

[17] Palleschi L (2014). Acute functional decline before hospitalization in older patients. Geriatr. Gerontol. Int..

[18] Gill TM, Gahbauer EA, Murphy TE, Han L, Allore HG (2012). Risk factors and precipitants of long-term disability in community mobility: A cohort study of older persons. Ann. Intern. Med..

[19] Hossain M, Yu D, Bikdeli B, Yu S (2021). Sarcopenia and adverse post-surgical outcomes in geriatric patients: A scoping review. J Frailty Aging.

[20] Humphry NA (2021). Association of postoperative clinical outcomes with sarcopenia, frailty, and nutritional status in older patients with colorectal cancer: Protocol for a prospective cohort study. JMIR Res. Protoc..

[21] Whittle J, Wischmeyer PE, Grocott MPW, Miller TE (2018). Surgical prehabilitation: Nutrition and exercise. Anesthesiol. Clin..

[22] Shen Y, Hao Q, Zhou J, Dong B (2017). The impact of frailty and sarcopenia on postoperative outcomes in older patients undergoing gastrectomy surgery: A systematic review and meta-analysis. BMC Geriatr..

[23] Rollins KE, Lobo DN (2016). Intraoperative goal-directed fluid therapy in elective major abdominal surgery: A meta-analysis of randomized controlled trials. Ann. Surg..

[24] Schwenk W (2022). Optimized perioperative management (fast-track, ERAS) to enhance postoperative recovery in elective colorectal surgery. GMS Hyg. Infect. Control.

[25] Melloul E (2016). Guidelines for perioperative care for liver surgery: Enhanced recovery after surgery (ERAS) society recommendations. World J. Surg..

[26] Carli F, Scheede-Bergdahl C (2015). Prehabilitation to enhance perioperative care. Anesthesiol. Clin..

[27] Topp R, Ditmyer M, King K, Doherty K, Hornyak J (2002). The effect of bed rest and potential of prehabilitation on patients in the intensive care unit. AACN Clin. Issues.

[28] Ljungqvist O (2014). ERAS—Enhanced recovery after surgery: Moving evidence-based perioperative care to practice. JPEN J. Parenter. Enter. Nutr..

[29] De Luca R (2022). Immunonutrition and prehabilitation in pancreatic cancer surgery: A new concept in the era of ERAS^®^ and neoadjuvant treatment. Eur. J. Surg. Oncol..

[30] Prehabilitation, rehabilitation, and revocation in the Army. *Br. Med. J.* **1**, 192–197. (1946). 20989832

[31] Lundberg M, Archer KR, Larsson C, Rydwik E (2019). Prehabilitation: The emperor’s new clothes or a new arena for physical therapists?. Phys. Ther..

[32] Michael CM, Lehrer EJ, Schmitz KH, Zaorsky NG (2021). Prehabilitation exercise therapy for cancer: A systematic review and meta-analysis. Cancer Med..

[33] Bausys A (2022). The role of prehabilitation in modern esophagogastric cancer surgery: A comprehensive review. Cancers.

[34] Lyons NB, Bernardi K (2020). Prehabilitation among patients undergoing non-bariatric abdominal surgery: A systematic review. J. Am. Coll. Surg..

[35] Hughes MJ (2019). Prehabilitation before major abdominal surgery: A systematic review and meta-analysis. World J. Surg..

[36] Thomas G, Tahir MR (2019). Prehabilitation before major intra-abdominal cancer surgery: A systematic review of randomised controlled trials. Eur. J. Anaesthesiol..

[37] Luther A, Gabriel J, Watson RP, Francis NK (2018). The impact of total body prehabilitation on post-operative outcomes after major abdominal surgery: A systematic review. World J. Surg..

[38] Hijazi Y, Gondal U, Aziz O (2017). A systematic review of prehabilitation programs in abdominal cancer surgery. Int. J. Surg..

[39] Heger P (2020). A systematic review and meta-analysis of physical exercise prehabilitation in major abdominal surgery (PROSPERO 2017 CRD42017080366). J. Gastrointest. Surg..

[40] Pang NQ (2022). Multimodal prehabilitation in older adults before major abdominal surgery: A systematic review and meta-analysis. Langenbecks Arch. Surg..

[41] Waterland JL (2021). Efficacy of prehabilitation including exercise on postoperative outcomes following abdominal cancer surgery: A systematic review and meta-analysis. Front. Surg..

[42] Daniels SL (2020). Prehabilitation in elective abdominal cancer surgery in older patients: Systematic review and meta-analysis. BJS Open.

[43] Yoshida N, Harada K, Iwatsuki M, Baba Y, Baba H (2020). Precautions for avoiding pulmonary morbidity after esophagectomy. Ann. Gastroenterol. Surg..

[44] Page MJ (2021). The PRISMA 2020 statement: An updated guideline for reporting systematic reviews. BMJ.

[45] Shea BJ (2017). AMSTAR 2: A critical appraisal tool for systematic reviews that include randomised or non-randomised studies of healthcare interventions, or both. BMJ.

[46] Amirkhosravi F (2023). OSF Regist..

[47] Search strategies. Accessed 22 February 2022. https://bestpractice.bmj.com/info/us/toolkit/learn-ebm/study-design-search-filters/. (2017).

[48] Glanville J, Dooley G, Wisniewski S, Foxlee R, Noel-Storr A (2019). Development of a search filter to identify reports of controlled clinical trials within CINAHL Plus. Health Info. Libr. J..

[49] Schardt C, Adams MB, Owens T, Keitz S, Fontelo P (2007). Utilization of the PICO framework to improve searching PubMed for clinical questions. BMC Med. Inform. Decis. Mak..

[50] Fried LP (2001). Frailty in older adults: Evidence for a phenotype. J. Gerontol. A Biol. Sci. Med. Sci..

[51] Peinemann F, Tushabe DA, Kleijnen J (2013). Using multiple types of studies in systematic reviews of health care interventions—A systematic review. PLoS One.

[52] Ditmyer MM, Topp R, Pifer M (2002). Prehabilitation in preparation for orthopaedic surgery. Orthop. Nurs..

[53] Arthur HM, Daniels C, McKelvie R, Hirsh J, Rush B (2000). Effect of a preoperative intervention on preoperative and postoperative outcomes in low-risk patients awaiting elective coronary artery bypass graft surgery: A randomized, controlled trial. Ann. Intern. Med..

[54] Covidence systematic review software, Veritas Health Innovation, Melbourne, Australia. Accessed 12 June 2024. www.covidence.org. (2020).

[55] Tufanaru C, Munn Z, Aromataris E, Campbell J, Hopp L, Aromataris E, Munn Z (2019). Chapter 3: Systematic reviews of effectiveness. JBI Reviewer’s Manual.

[56] Wan X, Wang W, Liu J, Tong T (2014). Estimating the sample mean and standard deviation from the sample size, median, range and/or interquartile range. BMC Med. Res. Methodol..

[57] Ryan R, Cochrane Consumers and Communication Review Group. Cochrane Consumers and Communication Group: meta-analysis. Accessed 20 January 2023. https://cccrg.cochrane.org/sites/cccrg.cochrane.org/files/public/uploads/meta-analysis_revised_december_1st_1_2016.pdf. (2016).

[58] Steffens D (2021). Feasibility and acceptability of a preoperative exercise program for patients undergoing major cancer surgery: Results from a pilot randomized controlled trial. Pilot Feasibility Stud..

[59] Richards T (2020). Preoperative intravenous iron to treat anaemia before major abdominal surgery (PREVENTT): A randomised, double-blind, controlled trial. Lancet.

[60] Dronkers JJ (2010). Preoperative therapeutic programme for elderly patients scheduled for elective abdominal oncological surgery: A randomized controlled pilot study. Clin. Rehabil..

[61] Barberan-Garcia A (2018). Personalised prehabilitation in high-risk patients undergoing elective major abdominal surgery. Ann. Surg..

[62] Boden I (2018). Preoperative physiotherapy for the prevention of respiratory complications after upper abdominal surgery: Pragmatic, double blinded, multicentre randomised controlled trial. BMJ.

[63] Dunne DFJ (2016). Randomized clinical trial of prehabilitation before planned liver resection. Br. J. Surg..

[64] Gillis C (2016). Prehabilitation with whey protein supplementation on perioperative functional exercise capacity in patients undergoing colorectal resection for cancer: A pilot double-blinded randomized placebo-controlled trial. J. Acad. Nutr. Diet..

[65] McIsaac DI (2022). Home-based prehabilitation with exercise to improve postoperative recovery for older adults with frailty having cancer surgery: The PREHAB randomised clinical trial. Br. J. Anaesth..

[66] de Soares SMTP, Nucci LB, da Silva MMC, Campacci TC (2013). Pulmonary function and physical performance outcomes with preoperative physical therapy in upper abdominal surgery: A randomized controlled trial. Clin. Rehabil..

[67] Ausania F (2019). Prehabilitation in patients undergoing pancreaticoduodenectomy: A randomized controlled trial. Rev. Esp. Enferm. Dig..

[68] Fernandez-Bustamante A (2017). Postoperative pulmonary complications, early mortality, and hospital stay following noncardiothoracic surgery: A multicenter study by the perioperative research network investigators. JAMA Surg..

[69] Merkow RP (2020). A comprehensive estimation of the costs of 30 day postoperative complications using actual costs from multiple, diverse hospitals. Jt. Comm. J. Qual. Patient Saf..

[70] Fleisher LA, Linde-Zwirble WT (2014). Incidence, outcome, and attributable resource use associated with pulmonary and cardiac complications after major small and large bowel procedures. Perioper. Med..

[71] Iacovazzo C (2023). Robot-assisted versus laparoscopic gastrointestinal surgery: A systematic review and metanalysis of intra- and post-operative complications. J. Pers. Med..

[72] Aiolfi A, Lombardo F, Bonitta G, Danelli P, Bona D (2021). Systematic review and updated network meta-analysis comparing open, laparoscopic, and robotic pancreaticoduodenectomy. Updates Surg..

[73] Ljungqvist O (2021). Opportunities and challenges for the next phase of enhanced recovery after surgery: A review. JAMA Surg..

[74] Fearon KCH (2005). Enhanced recovery after surgery: A consensus review of clinical care for patients undergoing colonic resection. Clin. Nutr..

[75] History. ERAS^®^ Society. Accessed 12 June 2024. https://erassociety.org/about/history/. (2016).

