# Supplementary material for: Multimodal prehabilitation and postoperative outcomes in upper abdominal surgery: systematic review and meta-analysis
Source: Sci Rep. 2024 Jul 11;14:16012. doi: 10.1038/s41598-024-66633-6 (PMC11239889; doi:10.1038/s41598-024-66633-6)
Supplement: Supplementary file 2 — Supplementary Information 2. [file 41598_2024_66633_MOESM2_ESM.pdf]

Article title: Multimodal Prehabilitation and Postoperative Outcomes in Upper Abdominal Surgery: Systematic Review and Meta-analysis

Supplementary Information

Contents

Supplementary File: Search Strategy 2

Supplementary Figure S1: Overall risk of bias using Joana Briggs Institute (JBI) assessment 4

Supplementary Figure S2: Risk of bias of included studies using JBI 5

Supplementary Figure S3: Postoperative Pulmonary Complications Funnel Plot 6

Supplementary Figure S4: All Postoperative Complications Funnel Plot 7

Sensitivity Analysis – Postoperative Complications 8

Sensitivity Analysis – Hospital Length of Stay 9

Supplementary File: Included_studies_outcome_measures.xlsx (see separate file)

Supplementary File: Search Strategy

**Medline (Ovid), 11/15/2021, updated 2/22/2022, 459 results total**
1. pancreatectomy/ or pancreaticoduodenectomy/ or pancreaticojejunostomy/
2. (Whipple or pancreatic enucleation or pancreatectomy or pancreaticoduodenectomy or pancreaticojejunostomy).ti,ab,kw.
3 ((elective adj3 (hepatobiliary or gastric or colon or abdominal) adj2 surger*) or (open adj2 hepatectomy)).ti,ab.
4. (elective adj2 surger*).ti,ab. or exp Elective Surgical Procedures/
5. exp Abdomen/ or exp Liver/ or exp Colon/ or (abdomen or abdominal or liver or colon or gastr*).ti,ab.
6. 1 or 2 or 3 or (4 and 5)
7. exp Preoperative Care/ or exp Exercise/ or exp Physical Therapy Modalities/ or exp Exercise Therapy/ or exp Diet Therapy/ or exp Psychotherapy/ or exp Cognitive Behavioral Therapy/
8. (rehab* or preoperat* or prehab*).fs,kw,ti,ab. or ((diet* or nutrit*) adj3 (therap* or intervent*)).ti,ab,kw. or ((exercise or physical) adj3 (therap* or intervent*)).ti,ab,kw. or ((psych* or behav) adj3 (therap* or intervent*)).ti,ab,kw.
9. 7 or 8
10. "randomized controlled trial".pt.
11. (random$ or placebo$ or single blind$ or double blind$ or triple blind$).ti,ab.
12. (retraction of publication or retracted publication).pt.
13. or/10-12
14. (animals not humans).sh.
15. ((comment or editorial or meta-analysis or practice-guideline or review or letter) not "randomized controlled trial").pt.
16. (random sampl$ or random digit$ or random effect$ or random survey or random regression).ti,ab. not "randomized controlled trial".pt.
17. or/14-16
18. 13 not 17
19. 6 and 9 and 18
20. limit 19 to yr="2000 -Current"

**Embase (Ovid) 2/22/2022, 776 results**
1. exp pancreatectomy/ or exp pancreaticoduodenectomy/ or exp pancreaticojejunostomy/
2. (Whipple or pancreatic enucleation or pancreatectomy or pancreaticoduodenectomy or pancreaticojejunostomy).ti,ab,kw.
3. ((elective adj3 (hepatobiliary or gastric or colon or abdominal) adj2 surger*) or (open adj2 hepatectomy)).ti,ab.
4. (elective adj2 surger*).ti,ab. or exp Elective Surgical Procedures/
5. exp Abdomen/ or exp Liver/ or exp Colon/ or (abdomen or abdominal or liver or colon or gastr*).ti,ab.
6. 1 or 2 or 3 or (4 and 5)
7. exp preoperative care/ or exp exercise/ or exp physiotherapy/ or exp kinesiotherapy/ or exp diet therapy/ or exp psychotherapy/ or exp cognitive behavioral therapy/
8. (rehab* or preoperat* or prehab*).fs,kw,ti,ab. or ((diet* or nutrit*) adj3 (therap* or intervent*)).ti,ab,kw. or ((exercise or physical) adj3 (therap* or intervent*)).ti,ab,kw. or ((psych* or behav) adj3 (therap* or intervent*)).ti,ab,kw.
9. 7 or 8
10. (random$ or placebo$ or single blind$ or double blind$ or triple blind$).ti,ab.
11. RETRACTED ARTICLE/

12.	10 or 11
13.	(animal$ not human$).sh,hw.
14.	(book or conference paper or editorial or letter or review).pt. not exp randomized controlled trial/
15.	(random sampl$ or random digit$ or random effect$ or random survey or random regression).ti,ab. not exp randomized controlled trial/
16.	12 not (13 or 14 or 15)
17.	6 and 9 and 16
18.	limit 17 to yr="2000 -Current"

**CINAHL (Ebsco), 2/22/2022, 266 articles**
S1 ((MH "Pancreatectomy") OR (MH "Pancreaticojejunostomy") OR (MH "Pancreaticoduodenectomy")) OR TI (Whipple or pancreatic enucleation or pancreatectomy or pancreaticoduodenectomy or pancreaticojejunostomy) OR AB (Whipple or pancreatic enucleation or pancreatectomy or pancreaticoduodenectomy or pancreaticojejunostomy)
S2 TI (((elective w3 (hepatobiliary or gastric or colon or abdominal) w2 surger*) or (open w2 hepatectomy))) OR AB (((elective w3 (hepatobiliary or gastric or colon or abdominal) w2 surger*) or (open w2 hepatectomy)))
S3 (MH "Surgery, Elective+") OR (AB elective w2 surger* OR TI elective w2 surger*)
S4 ((MH "Abdomen+") OR (MH "Liver") OR (MH "Colon+")) OR TI (abdomen or abdominal or liver or colon or gastr*) OR AB (abdomen or abdominal or liver or colon or gastr*)
S5 (S1 or S2) or (S3 and S4)
S6 ((MH "Preoperative Care+") OR (MH "Exercise+") OR (MH "Physical Therapy+") OR (MH "Therapeutic Exercise+") OR (MH "Diet Therapy+") OR (MH "Psychotherapy+") OR (MH "Cognitive Therapy+") OR (MH "Behavior Therapy+")) OR TI ((rehab* or preoperat* or prehab*) or ((diet* or nutrit*) w3 (therap* or intervent*)) or ((exercise or physical) w3 (therap* or intervent*)) or ((psych* or behav) w3 (therap* or intervent*))) OR AB ((rehab* or preoperat* or prehab*) or ((diet* or nutrit*) w3 (therap* or intervent*)) or ((exercise or physical) w3 (therap* or intervent*)) or ((psych* or behav) w3 (therap* or intervent*)))
S7 (randomized controlled trials OR MH double-blind studies OR MH single-blind studies OR MH random assignment OR MH pretest-posttest design OR MH cluster sample OR TI (randomised OR randomized) OR AB (random*) OR TI (trial) OR (MH (sample size) AND AB (assigned OR allocated OR control)) OR MH (placebos) OR PT (randomized controlled trial) OR AB (control W5 group) OR MH (crossover design) OR MH (comparative studies) OR AB (cluster W3 RCT)) NOT ((MH animals+ OR MH animal studies OR TI animal model*) NOT MH human)
S8 S5 and S6 and S7
Limit to 2000-current, no magazines

**Cochrane CENTRAL , 2/22/2022, 105, limited to Trials, and 2000-current**
#1 Whipple or pancreatic enucleation or pancreatectomy or pancreaticoduodenectomy or pancreaticojejunostomy
#2 ((elective adj3 (hepatobiliary or gastric or colon or abdominal) adj2 surger*) or (open adj2 hepatectomy))
#3 (elective adj2 surger*) and (abdomen or abdominal or liver or colon or gastr*)
#4 (rehab* or preoperat* or prehab*) or ((diet* or nutrit*) adj3 (therap* or intervent*)) or ((exercise or physical) adj3 (therap* or intervent*)) or ((psych* or behav) adj3 (therap* or intervent*))
#5 (#1 or #2 or #3) and #4
#6 accession near2 pubmed
#7 accession near3 embase
#5 not (#6 or #7)

Supplementary Figure S1: Overall risk of bias using Joana Briggs Institute (JBI) assessment

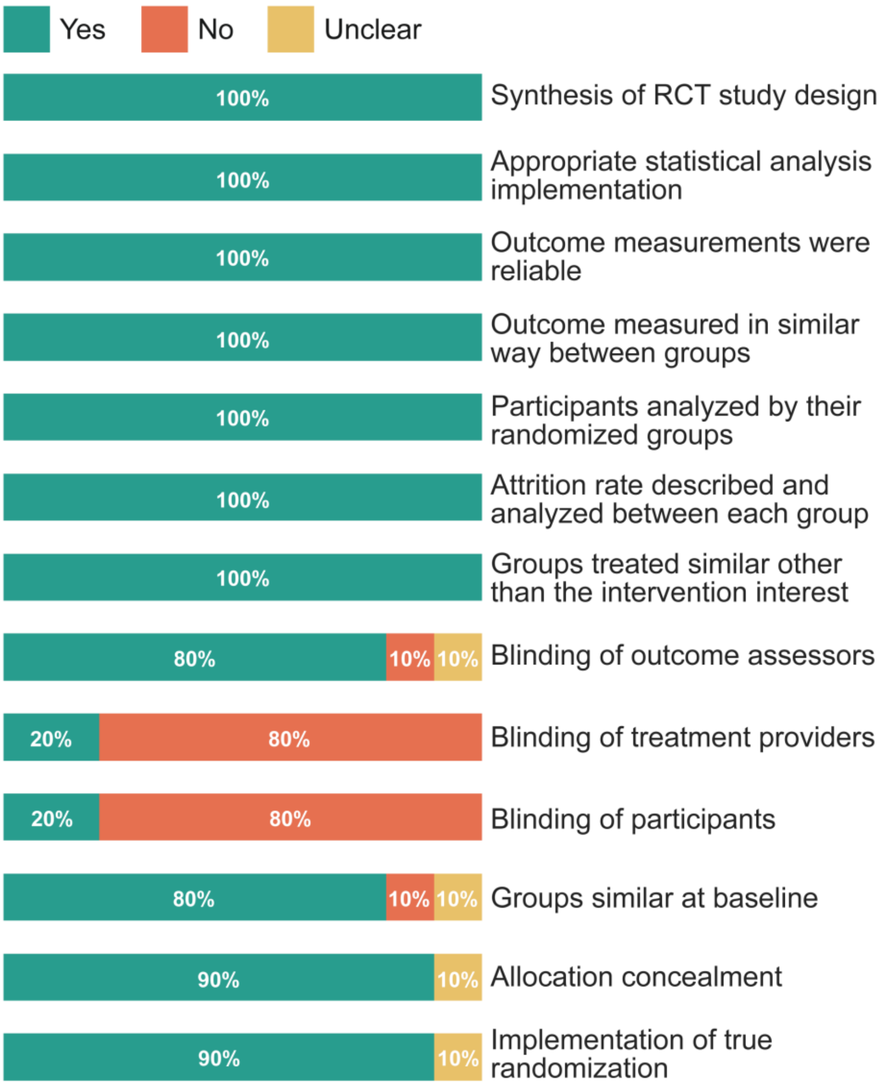

Supplementary Figure S2: Risk of bias of included studies using JBI

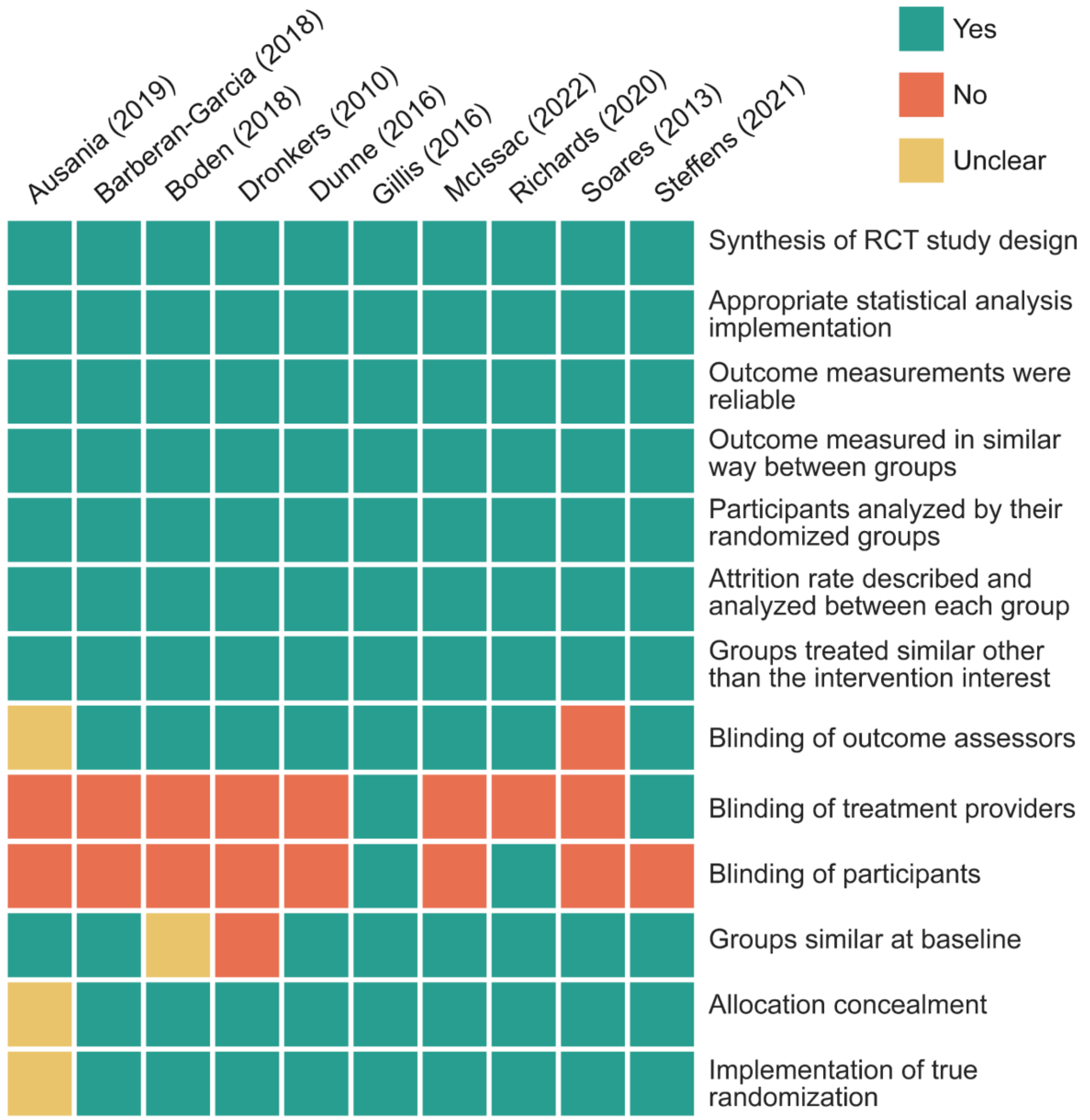

Supplementary Figure S3: Postoperative Pulmonary Complications Funnel Plot

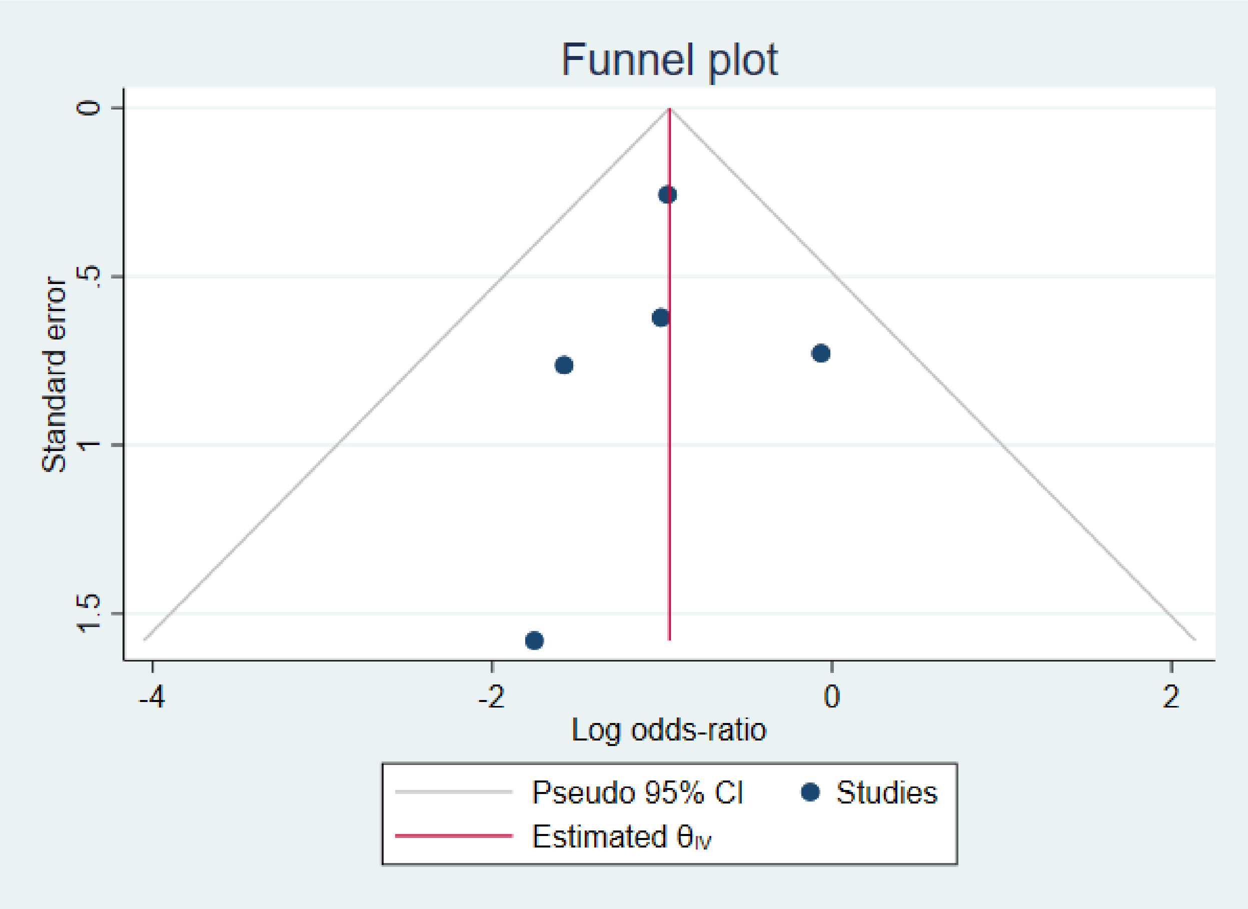

There was no significant heterogeneity (I2~0.0%). The overall log odds-ratio was -0.956 (-1.376 – -0.536, p<.0001) significantly. From funnel plot, we do not see potential publication bias.

Supplementary Figure S4: All Postoperative Complications Funnel Plot

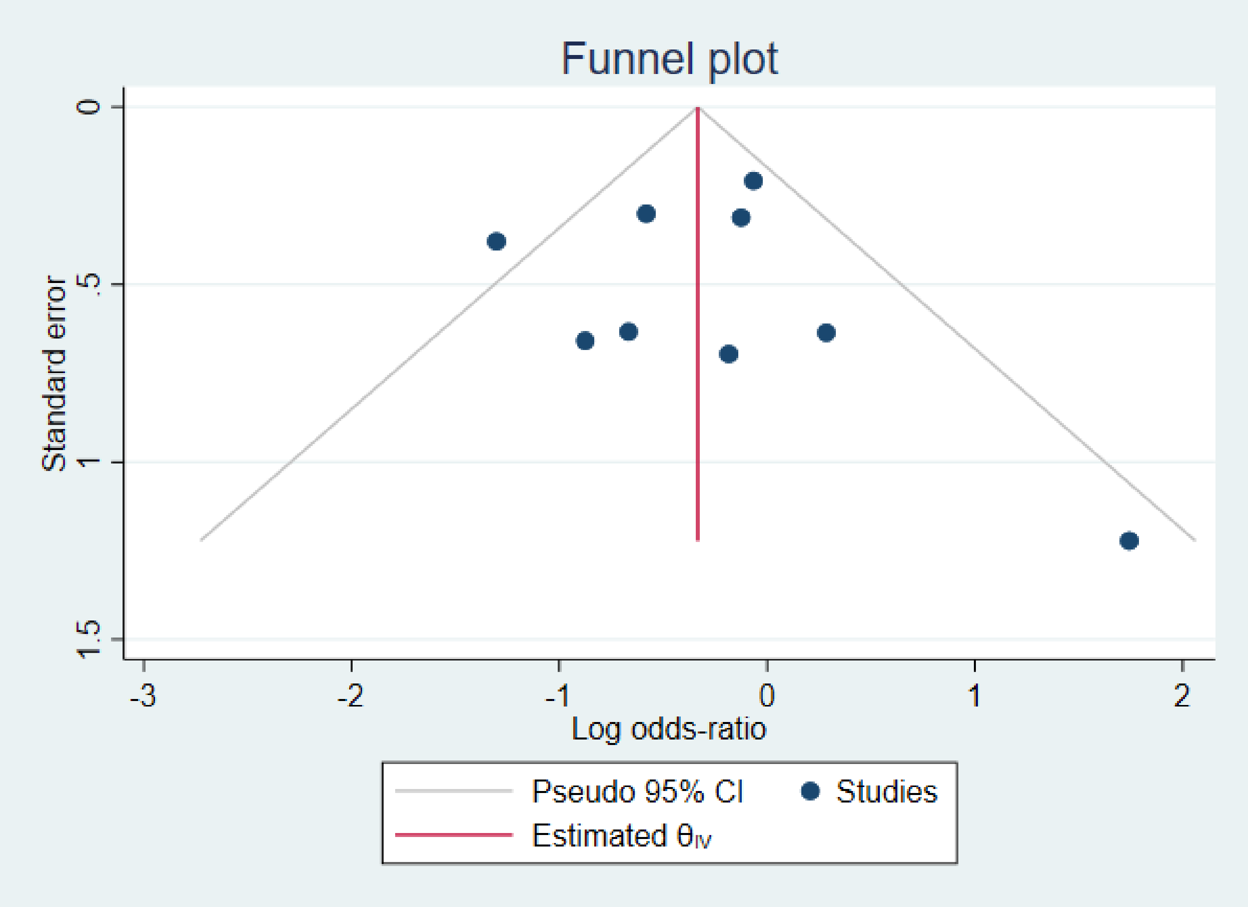

There was moderate heterogeneity (I2=41.06%) and potential publication bias with Barberan-Garcia, 2018. The overall log odds-ratio was -0.376 (-0.7449 – -0.004, p=0.0479) significantly.

Sensitivity Analysis – Postoperative Complications

We excluded Barberan-Garcia, 2018. The new model has low heterogeneity (I2=0.0%) and no potential publication bias. The overall log odds-ratio was -0.21 (-0.475 – 0.055, p=0.1209).

```
Meta-analysis summary                     Number of studies =      8
Random-effects model                      Heterogeneity:
Method: REML                                          tau2 =  0.0000
                                                    I2 (%) =    0.00
                                                        H2 =    1.00
```

Study	Log Odds-Ratio	[95% Conf.	Interval]	% Weight
Dronkers, 2010	0.285	-0.961	1.531	4.53
Dunne, 2016	-0.185	-1.548	1.178	3.78
Gillis, 2016	-0.667	-1.907	0.573	4.57
Boden, 2018	-0.065	-0.472	0.342	42.44
Ausania, 2019	-0.875	-2.166	0.415	4.22
Richards, 2020	-0.126	-0.735	0.484	18.91
Steffens, 2021	1.743	-0.652	4.138	1.23
McIsaac, 2022	-0.581	-1.169	0.007	20.32
theta	-0.210	-0.475	0.055	

```
Test of theta = 0: z = -1.55                     Prob > |z| = 0.1209
Test of homogeneity: Q = chi2(7) = 6.79           Prob > Q = 0.4505
```

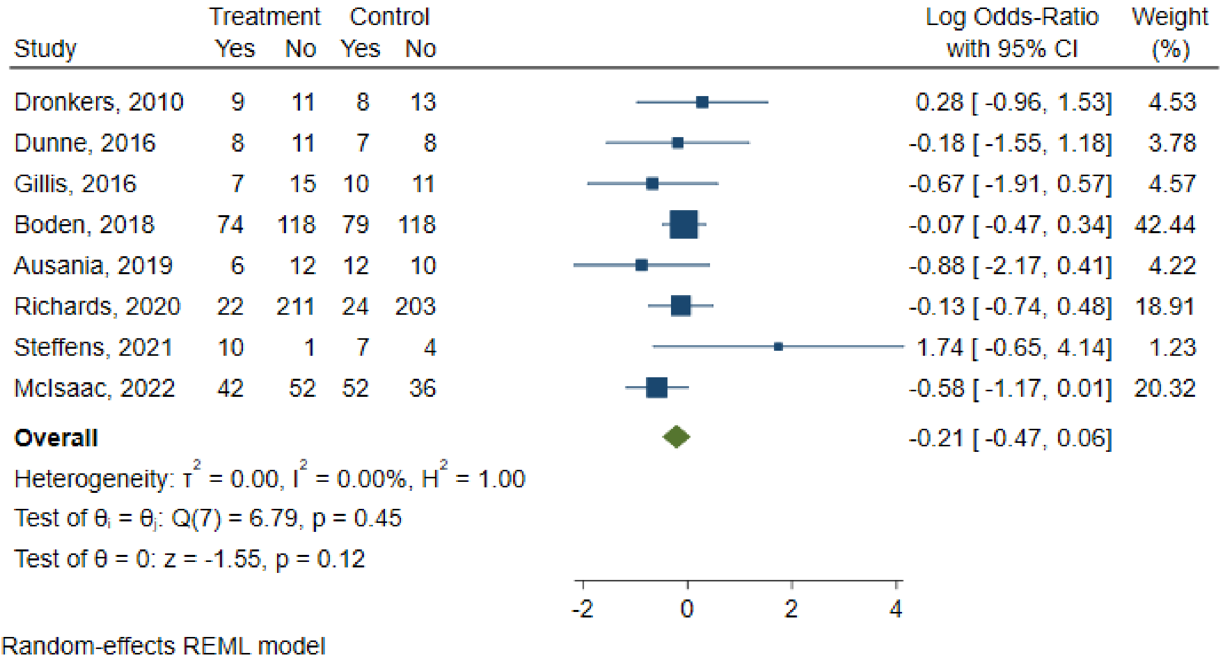

Sensitivity Analysis – Hospital Length of Stay

Study	Mean Diff.	[95% Conf.	Interval]	% Weight
Group: 0				
Gillis , 2016	1.333	-2.778	5.445	3.91
Richards, 2020	0.667	-0.401	1.734	22.94
theta	0.709	-0.325	1.742	
Group: 1				
Dronkers, 2010	-5.400	-16.716	5.916	0.57
Soares, 2013	-2.233	-7.470	3.003	2.51
Dunne, 2016	-0.500	-1.725	0.725	20.65
Barberan-Garcia, 2018	-5.000	-10.356	0.356	2.41
Boden, 2018	-1.333	-2.110	-0.557	27.51
Ausania, 2019	-5.267	-28.701	18.168	0.13
Steffens , 2021	2.000	-26.446	30.446	0.09
McIsaac, 2022	0.000	-1.325	1.325	19.27
theta	-0.906	-1.674	-0.138	
Overall				
theta	-0.478	-1.339	0.384	

Heterogeneity summary

Group	df	Q	P > Q	tau2	% I2	H2
0	1	0.09	0.758	0.000	0.00	1.00
1	7	6.62	0.469	0.186	15.48	1.18
Overall	9	14.31	0.112	0.546	39.60	1.66

Test of group differences: Q_b = chi2(1) = 6.04 Prob > Q_b = 0.014

Study	Treatment N	Treatment Mean	Treatment SD	Control N	Control Mean	Control SD	Mean Diff. with 95% CI	Weight (%)
**0**								
Gillis , 2016	22	7	7.924878	21	5.666667	5.565235	1.33 [-2.78, 5.44]	3.91
Richards, 2020	244	10	5.220226	243	9.333333	6.711885	0.67 [-0.40, 1.73]	22.94
Heterogeneity: τ^2 = 0.00, I^2 = 0.00%, H^2 = 1.00							0.71 [-0.32, 1.74]	
Test of $\theta_i = \theta_j$: Q(1) = 0.09, p = 0.76								
**1**								
Dronkers, 2010	21	16.2	11.5	20	21.6	23.7	-5.40 [-16.72, 5.92]	0.57
Soares, 2013	16	8.533333	6.096162	16	10.766667	8.778474	-2.23 [-7.47, 3.00]	2.51
Dunne, 2016	19	5	1.601938	15	5.5	2.044735	-0.50 [-1.72, 0.72]	20.65
Barberan-Garcia, 2018	62	8	8	63	13	20	-5.00 [-10.36, 0.36]	2.41
Boden, 2018	218	8.333333	3.731395	214	9.666667	4.478234	-1.33 [-2.11, -0.56]	27.51
Ausania, 2019	18	21.46667	31.37385	22	26.73333	42.00186	-5.27 [-28.70, 18.17]	0.13
Steffens , 2021	9	34.33333	32.36317	11	32.33333	32.23312	2.00 [-26.45, 30.45]	0.09
McIsaac, 2022	94	6.333333	3.764537	88	6.333333	5.275948	0.00 [-1.33, 1.33]	19.27
Heterogeneity: τ^2 = 0.19, I^2 = 15.48%, H^2 = 1.18							-0.91 [-1.67, -0.14]	
Test of $\theta_i = \theta_j$: Q(7) = 6.62, p = 0.47								
**Overall**							-0.48 [-1.34, 0.38]	
Heterogeneity: τ^2 = 0.55, I^2 = 39.60%, H^2 = 1.66								
Test of $\theta_i = \theta_j$: Q(9) = 14.31, p = 0.11								
Test of group differences: Q_b(1) = 6.04, p = 0.01								

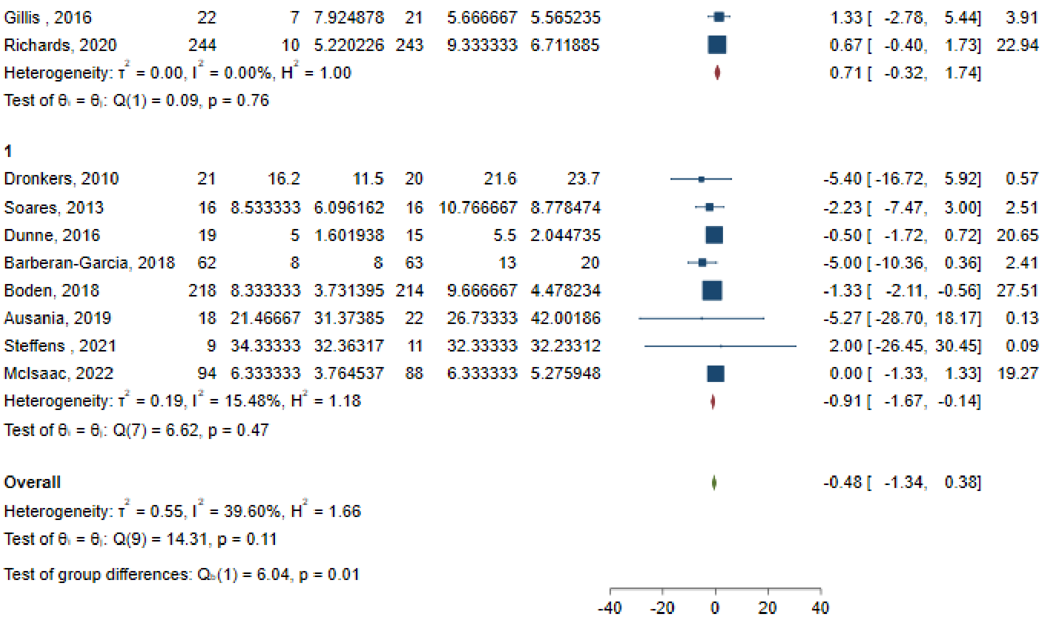

Random-effects REML model

After sub-grouping by prehab method (exercise vs. no exercise), the heterogeneity decreased to I2=15.48% in exercise group. In no exercise group, the LOS difference was not significant. In the exercise group, the difference was significantly -0.906 (-1.674 – -0.138, p=0.0208).
